# Innovative assessment of seasonal variations in body composition of elite soccer players with the integrated analysis DXA-BIVA

**DOI:** 10.1186/1550-2783-9-S1-P1

**Published:** 2012-11-19

**Authors:** Alessandro Bonuccelli, Andrea Causarano, Fulvio Marzatico, Saro Catanese, G D’Urbano, Stefano Beschi, Tim Ziegenfuss, D Buonocore, A Focarelli, Fabrizio Angelini

**Affiliations:** 1 Society of Sport Nutrition and Wellness (SINSeB), Italy; 2 AC Siena RoburTech, Siena, Italy; 3 Laboratory of Pharmacobiochemistry Nutrition and Nutriceuticals of Health, University of Pavia, Pavia, Italy; 4 The Center for Applied Health Sciences, Stow, Ohio, USA

## Background

Body composition (BC) and its changes over time may influence performance in soccer players. BC assessment techniques are mainly based on quantitative evaluation, originating from model-based indirect estimates of Fat-Free Mass and Fat Mass. DXA, particularly the advanced iDXA technology, is considered to be precise enough for this kind of assessment. On the other hand, Bio Impedance Vector Analysis (BIVA) allows the direct assessment of athletes’ body composition from impedance vector (Z vector), irrespective of body weight, prediction models or hydration assumptions and may classify qualitative changes in soft tissues hydration. The objective of this study was to investigate, compare, and integrate seasonal variations of soft tissues (assessed with DXA and BIVA) in elite soccer players, playing in the Italian top level championship (national major league).

## Methods

10 players (age 26.7 ± 3.) were evaluated throughout the championship. Fat-Free Mass and Fat Mass were assessed with DXA (Lunar iDXA, GE Healthcare). In the same time resistance and reactance components of impedance vector (Z vector) at 50 kHz frequency (BIA 101 RJL, Akern Italy) have been recorded. Measurements were performed at the beginning (T0) and at the end (T1) of the preseason training, therefore at mid (T2) and at the end (T3) of the regular season. During that period, athletes shared the same nutrition and supplementation programs.

## Results

From T0 to T1, FFM relative values increased significantly (82.2 ± 2.4% vs 85.1 ± 2.4% p<0.05) while FM% decreased considerably (13.8 ± 2.8% vs 10.8 ± 2.5%, p=0.55). Both values maintained steady during the rest of the season. Weight and BMI did not show significant changes during the whole period (p>0.05). Mean impedance vector placement differed significantly (Hotelling T^2^ test, p < 0.001), showing body water expansion and reduction respectively in T1 (compared to T0) and in T3 (compared to T1 and T2).

## Discussion

During the competitive season, athletes tested with both BIVA/iDXA techniques showed, as expected, an improvement of quantitative parameters of BC (Fat-Free Mass and Fat Mass) during the preseason period, and remaining almost unchanged during the rest of the season. However, parallel BIVA measurements show that early improvements of FFM/FM ratio were due to a mere fluid expansion, rather than a real change in muscle or lipid amount as DXA could wrongly display. In contrast, a sharp decrease of water compartment during the final stage of the season, against the same amount of Fat-Free Mass, during early- and mid-season period, suggests a possible improvement of muscle tissues during competitive season that DXA did not detect.

## Conclusion

According to our data, we found that DXA technique is not adequate to discriminate variations of the Fat-Free Mass protein/cellular and hydration components. We suggest therefore to complete soft tissues assessment with BIVA technique. DXA / BIVA methods should be considered as complementary, not alternative.

